# Estimating the proportion of metabolic health outcomes attributable to obesity: a cross-sectional exploration of body mass index and waist circumference combinations

**DOI:** 10.1186/s40608-016-0085-5

**Published:** 2016-01-29

**Authors:** Stephanie K. Tanamas, Viandini Permatahati, Winda L. Ng, Kathryn Backholer, Rory Wolfe, Jonathan E. Shaw, Anna Peeters

**Affiliations:** 1 Obesity and Population Health, Baker IDI Heart and Diabetes Institute, the Alfred Centre Level 4, 99 Commercial Road, Melbourne, VIC 3004 Australia; 2 Department of Epidemiology and Preventive Medicine, School of Public Health and Preventive Medicine, Monash University, the Alfred Centre Level 6, 99 Commercial Road, Melbourne, VIC 3004 Australia

## Abstract

**Background:**

Recent evidence suggests that a substantial subgroup of the population who have a high-risk waist circumference (WC) do not have an obese body mass index (BMI). This study aimed to explore whether including those with a non-obese BMI but high risk WC as ‘obese’ improves prediction of adiposity-related metabolic outcomes.

**Methods:**

Eleven thousand, two hundred forty-seven participants were recruited. Height, weight and WC were measured. Ten thousand, six hundred fifty-nine participants with complete data were included. Adiposity categories were defined as: BMI^N^/WC^N^, BMI^N^/WC^O^, BMI^O^/WC^N^, and BMI^O^/WC^O^ (N = non-obese and O = obese). Population attributable fraction, area under the receiver operating characteristic curve (AUC), and odds ratios (OR) were calculated.

**Results:**

Participants were on average 48 years old and 50 % were men. The proportions of BMI^N^/WC^N^, BMI^N^/WC^O^, BMI^O^/WC^N^ and BMI^O^/WC^O^ were 68, 12, 2 and 18 %, respectively. A lower proportion of diabetes was attributable to obesity defined using BMI alone compared to BMI and WC combined (32 % vs 47 %). AUC for diabetes was also lower when obesity was defined using BMI alone (0.62 vs 0.66). Similar results were observed for all outcomes. The odds for hypertension, dyslipidaemia, diabetes and CVD were increased for those with BMI^N^/WC^O^ (OR range 1.8–2.7) and BMI^O^/WC^O^ (OR 1.9–4.9) compared to those with BMI^N^/WC^N^.

**Conclusions:**

Current population monitoring, assessing obesity by BMI only, misses a proportion of the population who are at increased health risk through excess adiposity. Improved identification of those at increased health risk needs to be considered for better prioritisation of policy and resources.

**Electronic supplementary material:**

The online version of this article (doi:10.1186/s40608-016-0085-5) contains supplementary material, which is available to authorized users.

## Background

Obesity is estimated to be the third largest contributor to the overall burden of disease in Australia [1]. It is most commonly assessed using the body mass index (BMI) threshold of 30 kg/m^2^ or greater [2]. The limitations of BMI have been discussed in the literature, including in a review which suggested that it is time we move beyond the use of BMI to assess obesity to actual measurements of body fat mass [3]. However, methods such as underwater weighing, deuterium dilution, dual-energy X-ray absorptiometry, or bioelectrical impedance analysis reliably estimate total body fat, but are too expensive and time-consuming for population-level use.

Consequently, which anthropometric measure best identifies high-risk adiposity has been much debated. Various alternative measures to BMI have been advocated to improve estimates of adiposity and/or to provide better prediction of ill-health, including waist circumference (WC), waist-hip ratio, and waist-height ratio [4]. WC has generally been shown to be a better predictor of metabolic risk and chronic diseases than BMI. This is likely due to a combination of WC better identifying individuals at increased health risk through greater total body fat, and by also including those with central adiposity who are not necessarily identified by BMI [5, 6]. However, the degree of improvement in prediction of outcomes has not generally been seen as sufficient to drive a shift from BMI to WC.

New data has recently emerged suggesting that 40–70 % of the population classified as having high-risk WC have a BMI that is below the obese range [7–9]. However, relatively little is known about their associated health risks. In order to determine if they are a group that should be routinely identified as obese, it is important to know whether their health risks are greater than individuals with a non-obese BMI and non-obese WC. The aim of our study was to compare the metabolic health risks across different combinations of BMI and WC categories in a population of Australian adults, and to explore whether including those with a non-obese BMI but a high risk WC with those classified as ‘obese’ by BMI alone improves prediction of adiposity-related metabolic outcomes.

## Methods

### Study population

The Australian Diabetes, Obesity and Lifestyle (AusDiab) study methods and response rates have been described previously [10]. In brief, a stratified cluster sample of 11, 247 adults aged ≥25 years was drawn from 42 randomly selected census collector districts across Australia in 1999/2000. A total of 10, 659 participants were included in this analysis. 588 participants (5.2 %) were excluded due to missing data: BMI (*n* = 180); WC (*n* = 194); education (*n* = 110); country of birth (*n* = 2); smoking status (*n* = 212); TV viewing time (*n* = 92); CVD (*n* = 75); dyslipidaemia (*n* = 2); diabetes status (*n* = 169); and hypertension status (*n* = 73) (numbers are not additive). There was no difference in mean age between those with and without missing data. Women were more likely to have missing data than men (*p* < 0.001). This study was approved by the International Diabetes Institute Ethics Committee and the Monash University Human Research Ethics Committee. All participants gave written informed consent.

### Measurement of body mass index and waist circumference

Height was measured to the nearest 0.5 cm without shoes using a stadiometer. Weight was measured without shoes and excess clothing to the nearest 0.1 kg using a mechanical beam balance. BMI (kg/m^2^) was calculated as weight (kg) /height (m)^2^ and categorized as: (i) non-obese: <30 kg/m^2^; and (ii) obese: ≥30 kg/m^2^. WC was measured at the point midway between the iliac crest and the costal margin and the mean of two measures was calculated. WC was categorized as: (i) non-obese: <102 cm for men, <88 cm for women; and (ii) obese: ≥102 cm for men, ≥88 cm for women [6]. Adiposity categories were created using a combination of BMI and WC as follows: (i) BMI^N^/WC^N^; (ii) BMI^N^/WC^O^; (iii) BMI^O^/WC^N^; and (iv) BMI^O^/WC^O^, where N = non-obese and O = obese.

### Measurement of metabolic outcomes

In all states except Victoria, blood pressure was measured using a Dinamap® oscillometric blood pressure recorder (General Electric Company, Milwaukee, WI, USA). In Victoria, blood pressure was measured using a standard mercury sphygmomanometer and adjusted accordingly [11]. The average of two measures was used in the analysis. Hypertension was defined as blood pressure >140/90 mmHg or the use of antihypertensive medication.

Blood samples were collected by venepuncture after an overnight fast (≥9 h). All samples were centrifuged on-site to separate plasma and serum, and were transported daily to a central laboratory where possible. If transport to a central laboratory was not possible, samples were stored on-site in a freezer at −20 °C and then transferred to a −70 °C storage facility within 1 to 2 weeks following collection [12]. Diabetes was defined on the basis of fasting plasma glucose ≥7.0 mmol/l or two-hour plasma glucose ≥11.1 mmol/l, or current treatment with insulin or oral hypoglycaemic agents. Dyslipidemia was defined as triglycerides >2.0 mmol/l or high density lipoprotein (HDL) cholesterol <1.0 mmol/l. Cardiovascular disease (CVD) status was self-reported and was defined as previous angina, stroke, and/or coronary artery disease.

### Measurement of covariates

Covariate data was collected using interviewer-administered questionnaires. Educational attainment was categorized as: (i) low: secondary school qualification or lower; (ii) middle: attained trade or technician’s certificate, associate or undergraduate diploma, or nursing or teaching qualification; and (iii) high: attained a bachelor degree or post-graduate diploma. Country of birth was dichotomised into Europid and non-Europid. Physical activity was self-reported and was categorised as: (i) inactive (0 min/week); (ii) insufficient (1–149 min/ week); and sufficient (≥150 min/ week). Smoking status was categorised as: (i) current smoker; (ii) ex-smoker; and (iii) non-smoker. Time spent watching TV was used as a measure of sedentary behaviour and was categorised as: (i) <2 h/day; (ii) 2–3.9 h/day; and (iii) ≥4 h/day [13]. Information on alcohol consumption and energy intake were collected using the Food Frequency Questionnaire [14]. Alcohol consumption was dichotomised into ≤10 g/day and 10 g/day [15]. Energy intake was analysed as a continuous variable.

### Statistical analysis

To account for the clustering and stratification of the survey design, and to adjust for non-response, the data was weighted to match the age and sex distribution of the 1998 estimated residential population of Australia aged ≥25 years. The weighting factor was based on the probability of selection in each cluster.

Population attributable fraction (PAF) was calculated to determine the proportion of hypertension, diabetes, dyslipidaemia and CVD that is attributable to obesity, with obesity defined two ways: (i) obese according to either BMI or WC; and (ii) obese according to BMI alone. PAF was calculated using the formula:

$$ PAF={P}_E\frac{\left( OR-1\right)}{\left(1+{P}_E*\left( OR-1\right)\right)} $$

where P_E_ = prevalence of obesity and OR = odds ratio.

Discrimination for obesity defined as obese BMI or obese WC and obesity defined as obese BMI alone, with each metabolic outcome, was determined using area under the receiver operating characteristic curve (AUC) and compared using Wald chi-squared tests [16]. An AUC of 1.0 indicates perfect discrimination and AUC of 0.5 indicates that the discriminatory power of the predictor is no better than chance.

Logistic and linear regressions were used to explore the relationships of adiposity categories with hypertension, dyslipidaemia, diabetes and CVD, and with systolic blood pressure, fasting total cholesterol and fasting plasma glucose, respectively. Adjustments were made for age, sex, education, country of birth, alcohol consumption, smoking, and sedentary behaviour. Physical activity and energy intake were initially included in the multivariate models, however, they did not alter the relationship between adiposity measures and metabolic variables in this study and were excluded from the final model to avoid over adjustment [17]. An interaction term was considered for age (dichotomised using the approximate sample mean age as <55 years and ≥55 years) and sex with the adiposity categories to test whether the relationship between adiposity and our chosen metabolic variables differed by age or by sex. As an interaction was found with both age and sex, we analysed men and women, and those aged <55 and ≥55 years separately.

To test whether any differences between BMI and BMI plus WC classification was due to the larger proportion of individuals classified as obese using BMI plus WC compared to BMI alone, we shifted the BMI cut-point to define obesity, such that the same proportion of people would be identified as obese using BMI plus WC and using BMI alone. The proportion identified as obese using BMI plus WC (BMI ≥30 kg/m^2^ and/or WC ≥102 cm for men and ≥88 cm for women) was 32 %, thus we examined the alternate BMI cut-point of ≥28.5 kg/m^2^ which also identifies 32 % of the study population as obese.

All analyses were performed using STATA® 11.2 (STATA, college Station, TC, USA).

### Sensitivity analysis

To test for any potential effect of different BMI and WC cut-points for obesity for different countries of birth, a sensitivity analysis was performed excluding 1007 non-Europid participants.

## Results

**Fig. 1:**
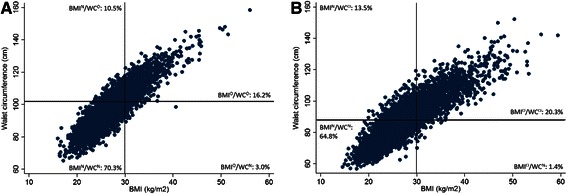
Prevalence of each adiposity category (BMI^N^/WC^N^: non-obese BMI and WC; BMI^N^/WC^O^: non-obese BMI, obese WC; BMI^O^/WC^N^: obese BMI, non-obese WC; BMI^O^/WC^O^: obese BMI and WC) in: **a** Men, and **b** women

The characteristics of the study population are presented in Table 1. The mean age of the participants was 48.2 years (SD 15.4 years) and 50 % were men. The majority of the population had their highest level of completed education as secondary school, and were Europid, sedentary and non-smokers. The prevalence of hypertension, diabetes, dyslipidaemia and CVD were 29, 7, 26 and 7 %, respectively. The mean BMI and WC were 26.6 kg/m^2^ and 90 cm (96 cm for men and 84 cm for women). Around two-thirds of the population were non-obese by both BMI and WC (BMI^N^/WC^N^), while 12, 2 and 18 % were categorised as BMI^N^/WC^O^, BMI^O^/WC^N^ and BMI^O^/WC^O^, respectively. Figure 1 presents the prevalence by sex.

**Table 1:** Characteristics of the study population

	Total	BMI^N^/WC^N^	BMI^N^/WC^O^	BMI^O^/WC^N^	BMI^O^/WC^O^
	*n = 10 659*	*n = 6 688*	*n = 1 594*	*n = 171*	*n = 2 206*
Age (years)	48.2 (15.4)	46.1 (14.6)	56.2 (17.4)	42.1 (10.9)	51.6 (15.2)
Male sex, %	50.0	52.0	43.5	68.0	44.3
Education, %					
Secondary school	37.0	33.7	44.2	35.1	44.7
Trade, technician’s certificate	32.2	32.1	31.9	33.5	32.8
Associate, undergraduate diploma,	11.7	12.3	10.6	11.0	10.5
nursing or teaching qualification					
Bachelor degree, post-graduate	19.1	21.9	13.4	20.4	11.9
Europid, %	87.5	86.3	92.1	84.5	89.4
BMI (kg/m^2^)	26.6 (4.9)	24.2 (2.7)	27.8 (1.9)	31.0 (0.8)	34.3 (4.2)
Waist circumference (cm)	90.0 (13.6)	83.6 (9.5)	98.4 (8.0)	94.4 (5.8)	107.5 (11.2)
TV viewing time, %					
<2 h/day	6.7	7.6	4.0	4.5	5.4
2- < 4 h/day	8.1	9.4	5.0	5.2	5.8
≥4 h/day	85.2	83.0	91.0	90.3	88.8
Alcohol consumption, ≤10 g/day (%)	62.1	59.4	64.5	63.6	70.5
Smoking status, %					
Current smoker	16.4	17.0	16.5	21.2	13.4
Ex-smoker	25.9	23.2	33.1	24.1	31.3
Non-smoker	57.8	59.8	50.3	54.7	55.3
Systolic blood pressure (mmHg)	128.1 (18.3)	124.8 (16.6)	133.8 (21.4)	126.8 (12.1)	136.6 (19.5)
Diastolic blood pressure (mmHg)	70.8 (11.7)	69.4 (11.0)	71.9 (13.8)	72.9 (7.5)	75.2 (12.2)
Fasting plasma glucose (mmol/l)	5.5 (1.1)	5.4 (0.8)	5.7 (1.2)	5.6 (0.8)	6.0 (1.7)
Total cholesterol (mmol/l)	5.5 (1.1)	5.4 (1.0)	5.8 (1.2)	5.7 (0.8)	5.8 (1.2)
Hypertension, %	28.9	20.8	45.0	19.0	49.7
Diabetes, %	7.0	3.7	10.7	4.0	17.2
Dyslipidaemia, %	25.9	18.4	36.2	28.0	46.6
Cardiovascular disease, %	7.1	5.4	13.4	2.3	9.9

Data presented as mean (standard deviation) unless otherwise specified

Adiposity categories: BMI^N^/WC^N^: non-obese BMI and WC; BMI^N^/WC^O^: non-obese BMI, obese WC; BMI^O^/WC^N^: obese BMI, non-obese WC; BMI^O^/WC^O^: obese BMI and WC

Hypertension defined as blood pressure ≥140/90 mmHg or on antihypertensive medication; diabetes defined as fasting plasma glucose ≥7.0 mmol/l or 2-hour plasma glucose ≥11.1 mmol/l, or on oral hypoglycaemic medication or insulin; dyslipidaemia defined as triglycerides ≥2.0 mmol/l or HDL cholesterol <1.0 mmol/l; CVD defined as previous angina, stroke and/or coronary artery disease

The PAFs indicate that 16 % of hypertension, 32 % of diabetes, 19 % of dyslipidaemia and 7 % of CVD was attributable to obesity, with obesity defined using BMI alone (Table 2). However, when obesity was defined using a combination of BMI or WC, the PAFs were significantly higher at 28, 47, 29 and 25 %, respectively. When stratified by sex and by age, the PAFs were generally higher in women compared to men, and in those aged <55 years compared to those aged ≥55 years (Additional file 1: Table S1). When we defined obesity using WC alone, the findings were similar to when obesity was defined using a combination of BMI or WC (data not shown).

**Table 2:** Population attributable fraction and area under the receiver operating characteristic curve for obesity defined using BMI and WC

	Obese by BMI or WC		Obese by BMI		Obese by alternative BMI	
	PAF (95 % CI)	AUC (95 % CI)	PAF (95 % CI)	AUC (95 % CI)	PAF (95 % CI)	AUC (95 % CI)
Hypertension	28.3 (24.4, 32.1)	0.62 (0.61, 0.63)	15.7 (13.2, 18.1)	0.57 (0.56, 0.58)	21.7 (19.1, 24.3)	0.59 (0.58, 0.60)
Diabetes	47.4 (41.0, 53.1)	0.66 (0.65, 0.68)	32.4 (26.4, 37.9)	0.62 (0.60, 0.64)	36.2 (28.8, 42.9)	0.63 (0.61, 0.65)
Dyslipidaemia	29.0 (25.2, 32.6)	0.64 (0.63, 0.65)	18.8 (15.9, 21.6)	0.60 (0.59, 0.61)	26.8 (23.2, 30.1)	0.63 (0.62, 0.64)
CVD	24.5 (16.0, 32.2)	0.58 (0.56, 0.60)	7.3 (−1.0, 14.8)	0.53 (0.51, 0.54)	14.0 (5.1, 22.1)	0.54 (0.53, 0.56)

Obese by BMI or WC: BMI ≥30 kg/m^2^ or WC ≥102 cm for men and ≥88 cm for women; obese by BMI: BMI ≥30 kg/m^2^; obese by alternative BMI: BMI ≥28.5 kg/m^2^

*PAF* population attributable fraction, *AUC* area under the receiver operating characteristic curve, *BMI* body mass index, *WC* waist circumference, *CVD* cardiovascular disease

Discrimination was significantly higher for obesity defined using a combination of BMI or WC compared to obesity defined using BMI alone (Table 2). Similar results were generally found when stratified by age and by sex, except for CVD in those aged <55 years and diabetes in those aged ≥55 years, where no significant difference in AUC between the two obesity definitions was observed (Additional file 1: Table S1).

**Fig. 2:**
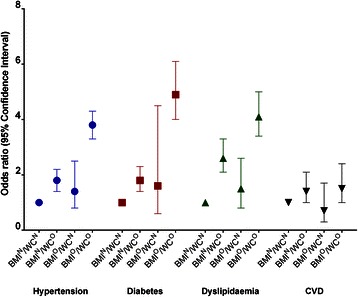
The relationship between adiposity categories (BMI^N^/WC^N^: non-obese BMI and WC; BMI^N^/WC^O^: non-obese BMI, obese WC; BMI^O^/WC^N^: obese BMI, non-obese WC; BMI^O^/WC^O^: obese BMI and WC) with hypertension, diabetes, dyslipidaemia and CVD, adjusted for age, sex, education, country of birth, TV viewing time, alcohol consumption and smoking status

Compared to those with BMI^N^/WC^N^, the odds for hypertension, diabetes and dyslipidaemia were increased in those with BMI^N^/WC^O^ and those with BMI^O^/WC^O^ (Fig. 2 and Additional file 2: Figure S1), with the greatest odds in the BMI^O^/WC^O^ group. In contrast, the odds for CVD were similar for both the BMI^N^/WC^O^ and BMI^O^/WC^O^ groups compared to those with BMI^N^/WC^N^. In all analyses, greater odds ratios were observed for the BMI^O^/WC^N^ group compared to the BMI^N^/WC^N^ group, though the results were not statistically significant. The magnitude of the odds ratios were generally higher in women and in those aged <55 years compared to men and those aged ≥55 years respectively. Systolic blood pressure, fasting plasma glucose and total cholesterol were elevated in both those with BMI^N^/WC^O^ and those with BMI^O^/WC^O^ compared to those with BMI^N^/WC^N^ (Table 3 and Additional file 3: Table S2). Total cholesterol, but not systolic blood pressure and fasting glucose, was also elevated in those with BMI^O^/WC^N^ compared to those with BMI^N^/WC^N^.

**Table 3:** Multivariate linear regression for associations of adiposity categories with systolic blood pressure, fasting plasma glucose and total cholesterol

	Adiposity categories			
	BMI^N^/WC^N^ Coefficient (Reference)	BMI^N^/WC^O^ Coefficient (95 % CI)	BMI^O^/WC^N^ Coefficient (95 % CI)	BMI^O^/WC^O^ Coefficient (95 % CI)
Systolic blood pressure (mmHg)	0.0	3.0 (1.5, 4.6)	3.3 (−0.5, 7.0)	8.7 (7.4, 10.0)
Fasting glucose (mmol/l)	0.0	0.2 (0.1, 0.3)	0.2 (−0.0, 0.4)	0.6 (0.5, 0.7)
Total cholesterol (mmol/l)	0.0	0.2 (0.1, 0.3)	0.4 (0.1, 0.7)	0.3 (0.3, 0.4)

Analyses were adjusted for age, sex, education, country of birth, TV viewing time, alcohol consumption and smoking status

Adiposity categories: BMI^N^/WC^N^: non-obese BMI and WC; BMI^N^/WC^O^: non-obese BMI, obese WC; BMI^O^/WC^N^: obese BMI, non-obese WC; BMI^O^/WC^O^: obese BMI and WC

When we used the alternative BMI cut-point for obesity (≥28.5 kg/m^2^), the proportion of the population with BMI^N^/WC^O^ decreased from 12 to 7 %, while the proportion with BMI^O^/WC^N^ and with BMI^O^/WC^O^ increased from 2 to 6 % and from 18 to 23 %, respectively. The proportion of hypertension, diabetes, dyslipidaemia and CVD attributable to obesity defined using the alternative BMI cut-point were higher compared to when obesity was defined using the conventional BMI cut-point of ≥30 kg/m^2^, though still lower than the proportions attributable to obesity defined using a combination of BMI or WC (Table 2 and Additional file 1: Table S1). Similarly, while the AUCs for obesity defined using the alternative BMI cut-point were higher compared to obesity defined using the conventional BMI cut-point, they were still significantly lower than the AUCs for obesity defined using a combination of BMI or WC (Table 2 and Additional file 1: Table S1).

In the sensitivity analysis, including only Europid participants did not significantly alter our results.

## Discussion

In a population of Australian adults aged 25 years and over, around one in three were classified as obese by their BMI or their WC. The PAF and AUC for each metabolic health outcome were higher when obesity was defined as having either an obese BMI or obese WC, indicating an improvement in the prediction of adiposity-related metabolic outcomes, compared to when defined as obese BMI alone. When compared to those who were non-obese (BMI^N^/WC^N^), we found increased odds for hypertension, diabetes, dyslipidaemia and CVD in those with BMI^N^/WC^O^ and those with BMI^O^/WC^O^. These findings highlight the importance of looking beyond BMI when assessing obesity in relation to health outcomes across the population, as even those with a BMI in the non-obese range may have elevated health risks that may be as high as those with an obese BMI.

Population attributable fraction estimates the proportion of disease that is attributable to a given risk factor, and thus represents the proportion of disease that theoretically would not have occurred had the risk factor not been present in the population [18]. In this study, we found that the proportion of metabolic outcomes attributable to an obese BMI was significantly lower than the proportion attributable to an obese BMI or WC combined. This addition of WC to BMI appeared to be beneficial as obesity defined using BMI alone had lower discriminative ability for outcomes compared to obesity defined using BMI and WC combined. Importantly, these differences persisted even after we lowered the BMI cut-point to increase the prevalence of the population classified as obese by BMI alone to the same as that identified by waist.

Our findings relating to obesity defined using BMI or WC combined and obesity defined using WC alone were very similar. However, we were unable to ascertain whether this reflects no added advantage in combining WC and BMI to define obesity rather than using WC alone, or whether this is because in this study, there was a modest number of participants with BMI^O^/WC^N^ (2 %), and therefore the individuals captured by defining obesity as either an obese BMI or WC is virtually the same as those captured by obese WC alone. Nevertheless, these findings further highlight the importance of looking beyond BMI when setting targets for the population. By incorporating WC to identify those with high risk adiposity and who should be targeted for intervention, the potential exists to prevent more metabolic outcomes in the population.

There has been little systematic analysis of the health risks associated with different combinations of BMI and WC. One study found that within each BMI category, those with a high-risk WC were more likely to have hypertension, diabetes, dyslipidaemia and the metabolic syndrome compared to those with a normal-risk WC [19]. This finding, while in line with ours, reported odds ratios but not absolute risks, and without absolute risk, it was not possible to determine whether the risk of disease in a person with high risk WC and obese BMI differed from a person with a high risk WC and non-obese BMI. To our best knowledge, only one previous study has found that, among individuals with a non-obese BMI, those with a high-risk WC (BMI^N^/WC^O^) had an increased risk for hypertension compared to those with a non-high risk WC (BMI^N^/WC^N^) [20]. Thus our study is among the first to demonstrate that by not regularly identifying those with obesity exclusively according to their WC, we are missing a group who appear to be at an increased risk of metabolic outcomes.

Previous studies that have compared different anthropometric measures in relation to health outcomes have generally concluded that while WC predicts metabolic outcomes more strongly that BMI, the improvements tend to be relatively modest and insufficient to warrant a move away from BMI to WC [21, 22]. More recently, there is evidence that BMI and WC have been tracking differently over the past few decades. In a number of populations, greater increases in WC than BMI have been observed, and WC has continued to increase despite an apparent plateau in BMI trends [8, 23–27]. The potential implication of these trends is that even if WC is only equivalent to or modestly better at predicting metabolic outcomes than BMI, WC may be increasingly capturing a different group of the population who have increased health risks related to excess adiposity than would be captured by BMI alone.

Taken together, our findings reinforce the importance of WC measurement in obesity assessment to accompany measures of BMI. Nevertheless, current population monitoring for obesity and identification of high risk individuals in clinical practice still tend to rely heavily on BMI [28, 29]. While some national weight management guidelines suggest that health professionals consider both BMI and WC, how to do so remains unclear within the guidelines [29]. Further, there are no similar recommendations for population health surveys. There needs to be a systematic move away from the sole use of BMI in obesity assessment towards identification of high risk adiposity based on WC or a combination of adiposity markers. Cut-points to identify high risk adiposity using any anthropometric measure are arbitrary since associations between risk factors and diseases are continuous, without discrete thresholds separating disease and no-disease. Nevertheless, cut-points are valuable for population health monitoring, to classify and quantify likely disease burdens, and to target health promotion.

A limitation to the current study is the small number of participants in the BMI^O^/WC^N^ category, which limited our ability to ascertain their odds for disease compared to those in other adiposity categories. Future studies in populations with a greater number of participants with BMI^O^/WC^N^ may help elucidate whether combining BMI and WC for obesity assessment would be of benefit, or whether simply moving to another single anthropometric measure, such as WC, is sufficient.

## Conclusion

In conclusion, one pitfall of current obesity monitoring is that it may be missing a large proportion of the population who have a high risk WC but a BMI below the obese range, who are at increased health risk through excess adiposity. Our findings support the need for a systematic approach to obesity assessment including identification of high risk adiposity based on other measures in addition to BMI. This will improve identification of those at increased risk of ill-health, which is important for better prioritisation of policy and resources.

## Additional files

Additional file 1: Table S1. Population attributable fraction and area under the receiver operating characteristic curve for obesity defined using BMI and WC (DOCX 16 kb)

Additional file 2: Figure S1. The relationship between adiposity categories (BMI^N^/WC^N^: non-obese BMI and WC; BMI^N^/WC^O^: non-obese BMI, obese WC; BMI^O^/WC^N^: obese BMI, non-obese WC; BMI^O^/WC^O^: obese BMI and WC) with hypertension, diabetes, dyslipidaemia and CVD in: A) men; B) women; C) age <55 years; and D) age ≥55 years; adjusted for age, sex, education, country of birth, TV viewing time, alcohol consumption and smoking status (TIFF 2978 kb)

Additional file 3: Table S2. Multivariate linear regression for associations of adiposity categories with systolic blood pressure, fasting plasma glucose and total cholesterol (DOCX 15 kb)

## Abbreviations

AUC: area under the receiver operating characteristic curve
AusDiab: Australian Diabetes, Obesity and Lifestyle
BMI: body mass index
CI: confidence interval
CVD: cardiovascular disease
HDL: high density lipoprotein
OR: odds ratio
PAF: population attributable fraction
SD: standard deviation
WC: waist circumference
