# Supplementary material for: Estimating the proportion of metabolic health outcomes attributable to obesity: a cross-sectional exploration of body mass index and waist circumference combinations
Source: BMC Obes. 2016 Jan 29;3:4. doi: 10.1186/s40608-016-0085-5 (PMC4734864; doi:10.1186/s40608-016-0085-5)
Supplement: Supplementary file 1 — Population attributable fraction and area under the receiver operating characteristic curve for obesity defined using BMI and WC (DOCX 16 kb) [file 40608_2016_85_MOESM1_ESM.docx]

Supplementary Table 1. Population attributable fraction and area under the receiver operating characteristic curve for obesity defined using BMI and WC

|  | Obese by BMI or WC | | Obese by BMI | | Obese by alternative BMI | |
| --- | --- | --- | --- | --- | --- | --- |
|  | PAF (95% CI) | AUC (95% CI) | PAF (95% CI) | AUC (95% CI) | PAF (95% CI) | AUC (95% CI) |
| *Men (n=4,830)* |  |  |  |  |  |  |
| Hypertension | 23.4 (18.0, 28.4) | 0.60 (0.59, 0.62) | 12.0 (8.6, 15.2) | 0.56 (0.54, 0.57) | 18.7 (14.8, 22.4) | 0.57 (0.56, 0.59) |
| Diabetes | 39.1 (30.8, 46.4) | 0.64 (0.62, 0.67) | 28.3 (19.6, 36.0) | 0.61 (0.59, 0.64) | 29.8 (24.2, 35.1) | 0.62 (0.60, 0.64) |
| Dyslipidaemia | 25.3 (21.2, 29.2) | 0.63 (0.61, 0.64) | 17.7 (13.7, 21.6) | 0.60 (0.59, 0.62) | 23.5 (19.9, 27.0) | 0.63 (0.61, 0.64) |
| CVD | 20.0 (10.2, 28.8) | 0.58 (0.55, 0.60) | 4.0 (-3.3, 10.9) | 0.52 (0.50, 0.54) | 13.4 (5.2. 21.0) | 0.55 (0.52, 0.57) |
| *Women (n=5,829)* |  |  |  |  |  |  |
| Hypertension | 34.3 (28.8, 39.4) | 0.65 (0.63, 0.66) | 19.7 (15.5, 23.7) | 0.59 (0.57, 0.60) | 25.0 (20.3, 29.4) | 0.60 (0.59, 0.61) |
| Diabetes | 59.4 (49.0, 67.7) | 0.69 (0.67, 0.71) | 37.9 (27.0, 47.2) | 0.63 (0.61, 0.66) | 43.4 (28.8, 55.0) | 0.64 (0.62, 0.67) |
| Dyslipidaemia | 39.5 (33.1, 45.3) | 0.67 (0.66, 0.69) | 22.2 (17.9, 26.3) | 0.60 (0.59, 0.62) | 31.0 (25.1, 36.5) | 0.64 (0.62, 0.65) |
| CVD | 29.7 (17.6, 40.0) | 0.59 (0.56, 0.62) | 10.5 (-1.4, 21.1) | 0.53 (0.51, 0.56) | 14.6 (1.8, 25.7) | 0.54 (0.51, 0.56) |
| *<55 years (n=6,489)* |  |  |  |  |  |  |
| Hypertension | 37.3 (28.8, 44.9) | 0.63 (0.61, 0.65) | 26.7 (22.6, 30.6) | 0.61 (0.59, 0.62) | 37.4 (31.4, 42.8) | 0.63 (0.61, 0.65) |
| Diabetes | 59.3 (48.5, 67.8) | 0.70 (0.67, 0.73) | 43.3(33.5, 51.6) | 0.67 (0.64, 0.71) | 51.4 (41.9, 59.4) | 0.69 (0.66, 0.72) |
| Dyslipidaemia | 31.7 (26.3, 36.7) | 0.64 (0.62, 0.65) | 20.5 (16.4, 24.4) | 0.61 (0.59, 0.62) | 29.5 (24.1, 34.5) | 0.64 (0.63, 0.66) |
| CVD | 32.0 (13.4, 46.6) | 0.62 (0.58, 0.67) | 20.4 (1.0, 36.1) | 0.59 (0.55, 0.63) | 34.6 (14.3. 50.1) | 0.62 (0.58, 0.66) |
| *≥55 years (n=4,170)* |  |  |  |  |  |  |
| Hypertension | 9.8 (6.3, 13.1) | 0.58 (0.57, 0.60) | 6.0 (4.5, 7.5) | 0.55 (0.54, 0.56) | 7.7 (5.6, 9.7) | 0.56 (0.55, 0.58) |
| Diabetes | 27.5 (20.2, 34.1) | 0.61 (0.59, 0.63) | 22.5 (16.2, 28.4) | 0.60 (0.58, 0.62) | 23.1 (14.5, 30.8) | 0.60 (0.58, 0.62) |
| Dyslipidaemia | 22.6 (16.4, 28.3) | 0.63 (0.61, 0.64) | 15.0 (11.9, 18.0) | 0.58 (0.57, 0.60) | 20.9 (15.7, 25.7) | 0.60 (0.59, 0.62) |
| CVD | 6.2 (-4.3, 15.6) | 0.53 (0.51, 0.54) | 0 (-7.3, 6.7) | 0.50 (0.48, 0.52) | 2.5 (-4.4, 9.0) | 0.51 (0.49, 0.53) |

PAF = population attributable fraction; AUC = area under the receiver operating characteristic curve; BMI = body mass index; WC = waist circumference; CVD = cardiovascular disease

Obese by BMI or WC: BMI ≥30 kg/m^2^ or WC ≥102 cm for men and ≥88 cm for women; obese by BMI: BMI ≥30 kg/m^2^; obese by alternative BMI: BMI ≥28.5 kg/m^2^
