# Supplementary figures and images for: Estimating the proportion of metabolic health outcomes attributable to obesity: a cross-sectional exploration of body mass index and waist circumference combinations
Source: BMC Obes. 2016 Jan 29;3:4. doi: 10.1186/s40608-016-0085-5 (PMC4734864; doi:10.1186/s40608-016-0085-5)

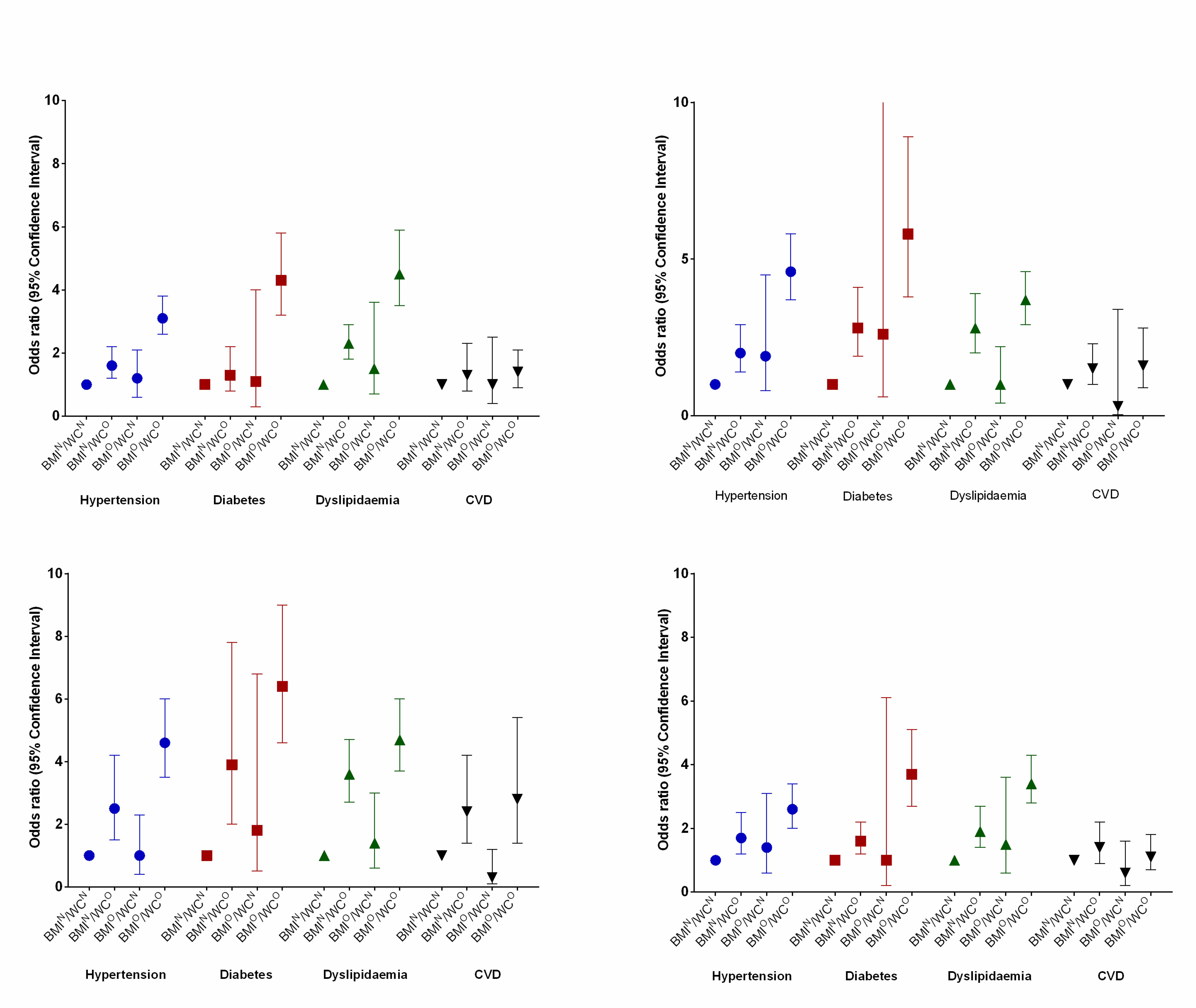

Supplement: Supplementary file 2 — The relationship between adiposity categories (BMIN/WCN: non-obese BMI and WC; BMIN/WCO: non-obese BMI, obese WC; BMIO/WCN: obese BMI, non-obese WC; BMIO/WCO: obese BMI and WC) with hypertension, diabetes, dyslipidaemia and CVD in: A) men; B) women; C) age <55 years; and D) age ≥55 years; adjusted for age, sex, education, country of birth, TV viewing time, alcohol consumption and smoking status (TIFF 2978 kb) [file 40608_2016_85_MOESM2_ESM.tiff]
