# Supplementary material for: Estimating the proportion of metabolic health outcomes attributable to obesity: a cross-sectional exploration of body mass index and waist circumference combinations
Source: BMC Obes. 2016 Jan 29;3:4. doi: 10.1186/s40608-016-0085-5 (PMC4734864; doi:10.1186/s40608-016-0085-5)
Supplement: Supplementary file 3 — Multivariate linear regression for associations of adiposity categories with systolic blood pressure, fasting plasma glucose and total cholesterol (DOCX 15 kb) [file 40608_2016_85_MOESM3_ESM.docx]

Supplementary Table 2. Multivariate linear regression for associations of adiposity categories with systolic blood pressure, fasting plasma glucose and total cholesterol

|  | Adiposity categories | | | |
| --- | --- | --- | --- | --- |
|  | BMI^N^/WC^N^ Coefficient (Reference) | BMI^N^/WC^O^ Coefficient (95% CI) | BMI^O^/WC^N^ Coefficient (95% CI) | BMI^O^/WC^O^ Coefficient (95% CI) |
| *Men (n=4,830)* | |  |  |  |
| Systolic blood pressure (mmHg) | 0.0 | 2.8 (1.1, 4.4) | 2.3 (-1.5, 6.1) | 7.4 (5.8, 9.0) |
| Fasting glucose (mmol/l) | 0.0 | 0.1 (-0.1, 0.2) | 0.2 (-0.0, 0.4) | 0.6 (0.4, 0.7) |
| Total cholesterol (mmol/l) | 0.0 | 0.2 (0.0, 0.4) | 0.3 (-0.1, 0.8) | 0.3 (0.2, 0.5) |
| *Women (n=5,829)* | |  |  |  |
| Systolic blood pressure (mmHg) | 0.0 | 3.4 (1.0, 5.8) | 4.0 (-1.0, 9.0) | 9.6 (7.9, 11.2) |
| Fasting glucose (mmol/l) | 0.0 | 0.3 (0.2, 0.4) | 0.3 (-0.2, 0.7) | 0.6 (0.5, 0.8) |
| Total cholesterol (mmol/l) | 0.0 | 0.2 (0.03, 0.3) | 0.4 (0.04, 0.7) | 0.3 (0.2, 0.4) |
| *<55 years (n=6,489)* | |  |  |  |
| Systolic blood pressure (mmHg) | 0.0 | 5.2 (3.2, 7.2) | 2.1 (-1.1, 5.4) | 10.8 (9.2, 12.4) |
| Fasting glucose (mmol/l) | 0.0 | 0.3 (0.2, 0.5) | 0.2 (0.1, 0.3) | 0.5 (0.4, 0.6) |
| Total cholesterol (mmol/l) | 0.0 | 0.3 (0.1, 0.5) | 0.3 (-0.0, 0.7) | 0.5 (0.4, 0.6) |
| *≥55 years (n=4,170)* | |  |  |  |
| Systolic blood pressure (mmHg) | 0.0 | 2.7 (-0.0, 5.5) | 2.9 (-5.4, 11.2) | 6.2 (3.3, 9.1) |
| Fasting glucose (mmol/l) | 0.0 | 0.1 (0.02, 0.3) | 0.2 (-0.6, 1.1) | 0.8 (0.6, 0.9) |
| Total cholesterol (mmol/l) | 0.0 | 0.1 (-0.0, 0.2) | 0.4 (-0.01, 0.7) | 0.1 (0.02, 0.2) |

Analyses were adjusted for age, sex, education, country of birth, TV viewing time, alcohol consumption and smoking status

Adiposity categories: BMI^N^/WC^N^: non-obese BMI and WC; BMI^N^/WC^O^: non-obese BMI, obese WC; BMI^O^/WC^N^: obese BMI, non-obese WC; BMI^O^/WC^O^: obese BMI and WC
